# ZIC1 modulates cell-cycle distributions and cell migration through regulation of sonic hedgehog, PI_3_K and MAPK signaling pathways in gastric cancer

**DOI:** 10.1186/1471-2407-12-290

**Published:** 2012-07-16

**Authors:** Jing Zhong, Shujie Chen, Meng Xue, Qin Du, Jianting Cai, Hongchuan Jin, Jianmin Si, Liangjing Wang

**Affiliations:** 1 Department of Gastroenterology, Second Affiliated Hospital, School of Medicine Zhejiang University, 88 Jiefang Road, Hangzhou, 310009, China; 2 Laboratory of Digestive Disease, Sir Run run Shaw Clinical Medicine Institution of Zhejiang University, 3 Qingchun Road, Hangzhou, 310053, China; 3 Key Laboratory of Biotherapy of Zhejiang Province, Biomedical Research Center, Sir Run Run Shaw Hospital, School of Medicine Zhejiang University, 3 Qingchun Road, Hangzhou, 310053, China

**Keywords:** ZIC1, Sonic hedgehog, Cell cycle, Tumour suppressor

## Abstract

**Background:**

ZIC1, a vital transcription factor with zinc finger domains, has been implicated in the process of neural development. We previously showed that ZIC1 may function as a tumour suppressor in gastrointestinal cancers. However, the molecular mechanism underlying ZIC1 participation in tumour progression remains unknown.

**Methods:**

The role of ZIC1 on cell proliferation and migration was examined. The regulation of sonic hedgehog (Shh), phosphoinositide 3-kinase (PI_3_K) and mitogen-activated protein kinase (MAPK) signaling pathways after ectopic expression of ZIC1 in gastric cancer cells were evaluated.

**Results:**

Overexpression of ZIC1 contributes to the inhibition of cell proliferation migration and cell-cycle distribution in gastric cancer. The modulation of G1/S checkpoint by ZIC1 is mainly mediated through the regulation of cyclin-dependent kinases (p21 ^Waf1/Cip1^, p27 ^Kip1^ and cyclin D1). In addition, ZIC1 can inactivate the level of phospholated Akt and Erk1/2, and transcriptionally regulate sonic hedgehog (Shh) signaling, thus leading to regulate the expression of p21 ^Waf1/Cip1^ and cyclin D1. Finally, we have systemically identified ZIC1 downstream targets by cDNA microarray analysis and revealed that 132 genes are down-regulated and 66 genes are up-regulated after transfection with ZIC1 in gastric cancer cells. These candidate genes play critical roles in cell proliferation, cell cycle and cell motility.

**Conclusions:**

Overexpression of ZIC1 results in inactivation of Shh, PI_3_K and MAPK signaling pathways, as well as regulation of multiple downstream targets which are essential for the development and progression of gastric cancer. ZIC1 serves as a potential therapeutic target for gastric cancer.

## Background

ZIC1, one of five ZIC family genes, is involved in a variety of developmental processes, including neurogenesis and myogenesis [1,2]. Recently, ZIC1 has been documented to participate in the progression of human tumours including medulloblastoma, endometrial cancers, mesenchymal neoplasms and liposarcoma cancers [3-6]. We have previously shown that ZIC1 gene is significantly downregulated in gastric cancer tissues and cell lines when compared with that of normal gastric tissues. In addition, ZIC1 potentially serves as a tumour suppressor by inhibiting cell proliferation in gastric and colorectal cancer cells [7,8]. As an important transcription factor, ZIC1 is essential to the regulation of Hedgehog signaling (Hh), Bone morphogenetic protein (BMP), and Notch signaling pathways in neural development [2,9]. However, little is known about how ZIC1 regulates signal pathways and their related downstream targets in cancer progression.

Gastric cancer is the second leading cause of cancer-related death worldwide [10,11]. Hh signaling is one of the key oncogenic signaling pathways involved in gastric carcinogenesis [12,13]. Sonic hedgehog (Shh), a member of the mammalian Hh family [14], has been demonstrated to be both upregulated in gastric cancer tissues by in situ hybridization assay and essential for the progression of gastric cancer [13,15]. Hh signaling pathway is activated by Shh binding with the Patched (Ptch)- Smoothened (Smo) membrane-receptor complex [16]. The activation of Shh promotes gastric cancer cell differentiation and proliferation. It has been reported that ZIC1 could reduce the expression of *PTCH1* and *Shh* genes in neural tissue during forebrain development [17]. In contrast, evidence has shown that the Shh represses the expression of ZIC1 during neural tube development [18]. Nevertheless, the influence of ZIC1 on the Hh signaling pathway in gastric cancer remains unknown.

ZIC1 also regulates numerous targets including cyclin D1, p27, Wnt1 and Wnt7a during neural development in xenopus ectodermal explants and mutant mice models [9,19,20]. We are particularly interested in cell-cycle regulators important for cancer cell proliferation and differentiation. We have previously shown that overexpression of ZIC1 can alter G1/S transition in gastric cancer cells [8]. Several mechanisms of Cyclin dependent kinases (CDKs) are involved in the regulation of G1/S checkpoint in human cancers [21]. During transition from G1 to S phase, cyclin D which forms active complexes with CDK4 is up-regulated in gastric cancer [22,23]. Loss of cyclin dependent kinase inhibitors such as p21^Waf1/Cip1^ and p27^Kip 1^ promote CDK2 activity and regulate the G1/S transition [12,24]. Recent studies have demonstrated that mutant ZIC1 or force overexpression of ZIC1 regulates the expression of p27 ^Kip 1^ in mice cerebellar tissues and liposarcoma cells [3,20]. Interestingly, the Shh signaling pathway negatively regulates p21 ^Waf1/Cip1^ and cyclin D1 in a GLI1-dependent manner [16,25]. However, whether ZIC1 can interplay with Shh pathway in the regulation of cell cycle distributions has not been defined. Elucidation of this signaling network may provide further insight into the role of ZIC1 in gastric cancer.

In our present study, we demonstrate that overexpression of ZIC1 suppresses gastric cancer cell migration and invasion, as well as alters the cell-cycle distributions. ZIC1 can transcriptionally downregulate the Shh signaling and suppress the level of phospholated Akt and Erk, thus leads to the regulation of cell-cycle regulator kinases p21^Waf1/Cip1^, p27^Kip1^ and cyclin D1 in gastric cancer cells. We also identified multiple important ZIC1 downstream targets in gastric cancer cells by cDNA microarray analysis.

## Results

### ZIC1 inhibits proliferation, migration and invasion of gastric cancer cells

**Figure 1:**
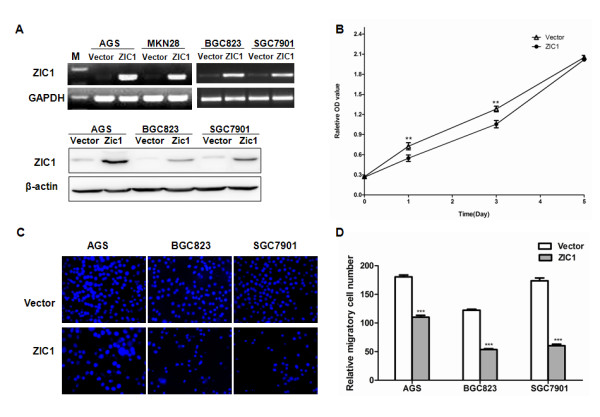
**ZIC1 suppresses cell proliferation and migration in gastric cancer.** (**A**) Gastric cancer cell lines (AGS, MKN28, BGC823 and SGC7901) were stably transfected with pCDNA3.1-ZIC1 or pCDNA3.1 empty vector. The expression levels of ZIC1 mRNA and protein were performed by RT-PCR (**upper diagram**) and Western blot (**low diagram**). GAPDH and β-actin were used as internal controls. (**B**) The cell viability of gastric cancer cell line (BGC823) was determined by MTS cell proliferation assay. The asterisk indicates statistical significance (**p < 0. 01). (**C**) Cell migration was assessed by modified Boyden transwell chambers assays after incubation for 16 h. Cells that migrated to the bottom of the membrane were stained with DAPI. (**D**) The number of visible migratory cells (mean ± S.D) was estimated by counting five random high power fields (×400 magnification). These experiments were performed in triplicate. The representative data are shown. The asterisk indicates statistical significance (***p < 0.001).

To determine the effect of ZIC1 on cell proliferation, we performed cell viability analysis by MTS assays in gastric cancer cells. Gastric cancer cell lines (AGS, MKN28, BGC823 and SGC7901) were transfected with pCDNA3.1-ZIC1 or pCDNA3.1 empty vector. The transfection efficiency was confirmed by RT-PCR and western blot respectively (Figure 1A). Results showed that the number of viable cells was significantly suppressed by ectopic expression of ZIC1 in a 5-day observation in BGC823 cells (Figure 1B). The suppression of cell proliferation by ZIC1 was consistent with our previous observations in AGS and MKN28 gastric cancer cells, as well as colon cancer cells [7,8].

In addition, we determined the role of ZIC1 in cell migration and invasion in gastric cancer. Cell migration and invasion assays were performed in transwell migration and Matrigel-coated invasion assay systems, respectively. We observed that re-expression of ZIC1 significantly suppressed cell migration in AGS, BGC823 and SGC7901 gastric cancer cell lines (p < 0.001) (Figure 1C,D). In addition, re-expression of ZIC1 displayed a significantly lower activity of cellular invasion when compared to those empty vector transfectants in AGS cells (p < 0.05) (Additional file 1: Figure S1). These data suggest that ectopic expression of ZIC1 suppresses gastric cancer cell migration and invasion.

### ZIC1 alters cell-cycle distributions and regulates the expression of cyclin-dependent kinases in gastric cancer cells

**Figure 2:**
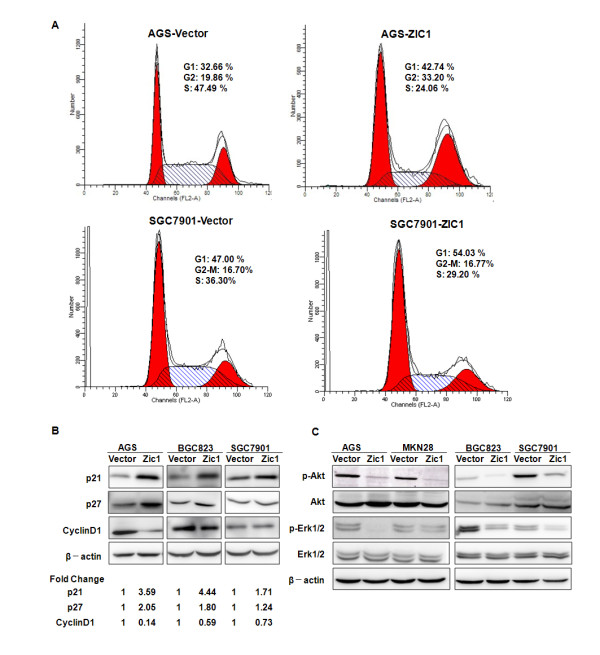
**ZIC1 alters cell-cycle distributions by regulation of p21, p27 and cyclin D1 in gastric cancer cells.** (**A**) Cell cycle distributions were detected by flow cytometry analysis in AGS and SGC7901 cells after transient transfection with pCDNA3.1-ZIC1 or pCDNA3.1 empty vector for 24 h. (**B**) The expression levels of p21, p27 and cyclin D1 were determined by Western blot. β-actin was detected as a loading control. Densitometry values are expressed as fold change compared with pCDNA3.1 vector control values normalized to 1. (**C**) The expression levels of phospholated Akt and Erk1/2, as well as total Akt and Erk1/2 were examined by Western blot.

To further understand the mechanisms underlying the inhibition of cell proliferation by overexpression of ZIC1, we evaluated cell-cycle distributions in gastric cancer cells. We observed a higher proportion of cells in G1 phase in AGS (42.74 %) and SGC7901 (54.03 %) cell lines after overexpression of ZIC1. However, in the cells transfected with a control vector, the proportion was decreased both in AGS (32.66 %) and SGC7901 (47.00 %) and the proportion of cells in S phase was relatively increased (Figure 2A). It is well accepted that p21 (also known as p21^Waf1/Cip1^) and p27 (also known as p27^KIP1^), two main cyclin-dependent kinase inhibitors, are required for cessation during the entry to S-phase [26]. The activation of Cyclin D1, however, is mainly responsible for regulating the G1-S phase transition [23]. We demonstrated that the expression level of cyclin D1 protein was reduced while p21 and p27 were markedly induced in gastric cancer cells transfected with pCDNA3.1-ZIC1 when compared to those pCDNA3.1 empty vector transfectants (Figure 2B). We also evaluated the cell apoptosis distributions in pCDNA3.1-ZIC1 or pCDNA3.1 vector transfectants in AGS and MKN28 cells, but no obvious differences of cell apoptosis were observed (Additional file 2: Figure S2). Therefore, these results support that overexpression of ZIC1 alters the cell cycle distributions through regulation of cyclin-dependent kinases p21, p27 as well as cyclin D1 in gastric cancer cells.

MAPK and PI_3_K pathways play vital roles in the regulation of cell-cycle kinases. We evaluated the expression of main downstream effectors of these two pathways, Erk1/2 and Akt, after stably introducing pCDNA3.1-ZIC1 to AGS, MKN28, BGC823 and SGC7901 gastric cancer cells (Figure 1A). We found that the phosphorylation levels of Erk1/2 and Akt were dramatically suppressed by overexpression of ZIC1 in all above cell lines tested (Figure 2C). These results suggested that the regulation of cell-cylce distribution by ZIC1 may be mediated through PI_3_K and MAPK pathways and their downstream cyclin-dependent kinases p21, p27 and cyclin D1 in gastric cancer.

### ZIC1 suppresses the expression of Sonic hedgehog (Shh) in gastric cancer cells

**Figure 3:**
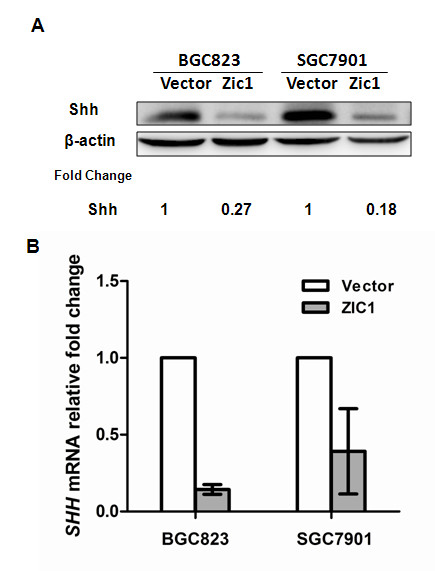
**ZIC1 inhibits the expression of Sonic hedgehog (Shh).** (**A**) The expression of Shh protein was analyzed by Western blot in BGC823 and SGC7901 cells stably transfected with pCDNA3.1-ZIC1 or pCDNA3.1 empty vector. (**B**) The expression level of Shh mRNA was determined by real-time quantitative RT-PCR analysis. The relative expression level was expressed as fold change (mean ± SEM) compared with pCDNA3.1 empty vector normalized to 1.

Molecular characterization has shown that *ZIC1* has zinc-finger domains and could counteract with GLI1 by binding to GC-rich sequences [2]. GLI1 is a downstream target of hedgehog (hh) signaling pathway which is essential to gastric cancer development and progression [9,27]. We hypothesized that Hh signaling pathway may be involved in the ZIC1 regulation of gastric cancer cell-cycle and cell migration. To address this issue, we examined the expression of Sonic hedgehog (Shh), a key member of Hh family, in gastric cancer cells after overexpression of ZIC1. We found that re-expression of ZIC1 effectively reduces Shh expression in BGC823 and SGC7901 cell lines by western blot analysis (Figure 3A). In addition, the RT-PCR analysis demonstrated that the transcript level of Shh is also significantly downregulated in gastric cancer cells transfected with ZIC1 relative to empty vector transfectants (p < 0.01) (Figure 3B) .( Additional file 3: Figure S3). Taken together, our results suggest that ZIC1 may transcriptionally regulate the expression of Shh in gastric cancer cells.

### Hedgehog (Hh) signaling pathway is involved in the ZIC1 regulation of cell-cycle and cell migration in gastric cancer cells

**Figure 4:**
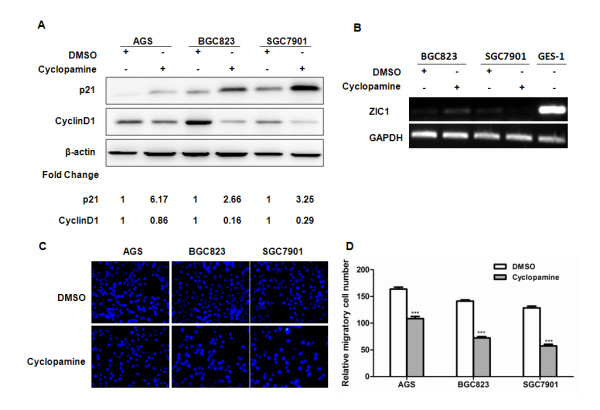
**Hh signaling pathway is involved in the expression of p21, cyclin D1 and regulation of cell migration.** AGS, BGC823 and SGC7901 gastric cancer cells were treated with cyclopamine (10 μM), a steroidal alkaloid that interacts directly with Smo to inhibit Hh signaling, or DMSO control for 24 h. (**A**) The expression levels of p21 and cyclin D1 were determined by western blot in AGS, BGC823 and SGC7901 cells. (**B**) The expression level of ZIC1 mRNA was examined through conventional RT-PCR. (**C**) Cell migration was assessed by modified Boyden transwell chambers assays. Cells that migrated to the bottom of the membrane were stained with DAPI. (**D**) The mean number of visible migratory cells (mean ± S.D) was calculated in five random high power fields (×400). The asterisk indicates statistical significance (***p < 0.001).

To determine the effect of Shh on cell-cycle distributions, we sought to examine the effects of pharmacologic inhibitor of Hh signaling on the expression of p21 and cyclin D1. AGS, BGC823 and SGC7901 gastric cancer cell lines were treated with cyclopamine (10 μM), a steroidal alkaloid that interacts directly with Smo to inhibit Hh signaling [28], or DMSO control for 24 h. We observed that the expression level of p21 was markedly up-regulated, while cyclin D1 down-regulated after tumour cells were treated with cyclopamine (Figure 4A). Of note, blocking the Shh signaling pathway by administration of cyclopamine does not affect the expression levels of ZIC1 mRNA in BGC823 and SGC7901 cells by RT-PCR assays (Figure 4B). The absent or low expression of ZIC1 mRNA in gastric cancer cells was mainly meditated by promoter DNA methylation as we described previously [8].

We also evaluated the effects of Shh signaling on gastric cancer cell migration. As shown in Figure 4 C and D, AGS, BGC823 and SGC7901 gastric cancer cell lines showed significant decrease in cellular migration after administration with cyclopamine (10 μM) for 24 h (p < 0.01). Collectively, these results demonstrate that ZICI may modulate the cell-cycle regulators and cell migration through Shh signaling in gastric cancer cells.

### Gene expression profile changes by ectopic expression of ZIC1

**Figure 5:**
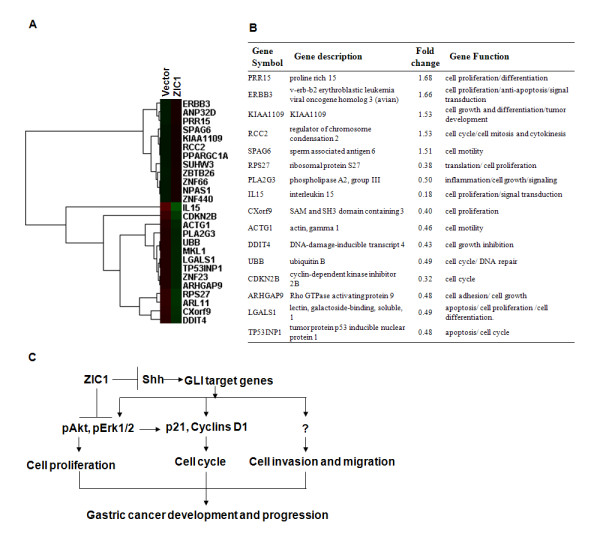
**Gene expression profile changes after ectopic expression of ZIC1.** (**A**) The gene expression profiles in pCDNA3.1-ZIC1 or empty pCDNA3.1 vector stable transfectants were analyzed by cDNA microarray in MKN28 cells. Representative genes are illustrated on the right side of the heatmap image using a cut-off >1.5 or < −1.5 fold as a significant difference,. (**B**) Genes involved in cell cycle, cell proliferation and cell migration according to gene functional analysis (http://www.genecards.org) are presented. Relative fold change is expressed with ratio of pCDNA3.1-ZIC1 versus pCDNA3.1 control. (**C**) Systemic understanding of pathways for ZIC1 regulation of signaling and target genes in the development and progression of gastric cancer.

To systematically determine downstream targets of ZIC1, we conducted an affymatrix oligonucleotide microarray in MKN28 gastric cancer cells with or without pCDNA3.1-ZIC1. Using a cut-off of >1.5 fold for statistical significance, a microarray revealed that 132 genes are down-regulated while 66 genes are up-regulated by exogenous expression of ZIC1 (representative genes shown in Figure 5A). Many genes have been reported to play critical roles in cell proliferation, cell cycle and cell migration according to gene functional analysis (http://www.genecards.org) (Figure 5B). For instance, two key cell-cycle regulators *TP53INPI* and *CDKN2B* are found to be deregulated in MKN28 cells tranfected with pCDNA3.1-ZIC1. Our results indicate that ZIC1 potentially regulates multiple downstream genes involved in gastric tumorigenesis.

## Discussion

Growing evidence has shown that ZIC1 is involved in the progression of several tumours [3-8]. It appears that ZIC1 is aberrantly expressed in certain types of cancer and differentially functions as a tumour suppressor or oncogenic gene. For instance, ZIC1 expression was reported to be low or absent in gastrointestinal and lung cancer cell lines, and was found to suppress gastrointestinal cancer cell proliferation [7,8,29]. In contrast, overexpression of ZIC1 in liposarcoma was found to promote cell proliferation and invasion [3]. We and others have demonstrated that the epigenetic modulations including DNA methylation and histone remodeling, and genetic mutations may contribute to its differential expression patterns in cancers [3,4,7,8]. It is becoming clear that as a zinc finger transcription factor, ZIC1 may modulate multiple downstream genes in neural tissue, colorectal cancer and liposarcoma cells [2,3,7,9]. However, little is known about the mechanism underlying ZIC1 function in the development and progression of gastric cancer. Underscoring the key pathways and downstream targets regulated by ZIC1 may facilitate our understanding of its roles in tumorigenesis. Here, we have demonstrated that overexpression of ZIC1 results in significant inhibition of cell survival and impairment of cell migration. ZIC1 suppresses the Shh , PI3K and MAPK signaling pathways which are critical for the regulation of cell-cycle distributions and cell migration in gastric cancer. Moreover, ZIC1 inhibits cell cycle regulatory kinases, p21, p27 and cyclin D1, thus leading to G1/S cell cycle transit in gastric cancer cells. ZIC1 suppresses the Shh signaling pathway which is critical for the regulation of cell-cycle distributions and cell migration in gastric cancer.

MAPK and PI_3_K pathways play critical roles in cell proliferation, differentiation, and progression in a variety of human cancers [30]. Recently, it was also reported that the activation of both pathways are essential to induce cell cycle entry [30,31]. During this process, cyclin dependent kinase inhibitors p21, p27 and cyclin D/E participate in the regulation of p53 induced cell-cycle arrest [24,32]. The activation of MAPK and its downstream kinase-Erk could not only lead to the induction of cyclin D1 and pass through G1/S checkpoint, but also the accumulation of p21 that inhibits cyclin E/CDK2 complexes to block S-phase entry [30]. PI_3_K/Akt pathway could inactivate the Gsk3-β and FOXO transcription factors, thus inhibit cyclin D1 while induce p27 and p21 in the regulation of cell-cycle entry [31]. We have shown that re-expression of ZIC1 effectively inactivate the phosphorylated Akt and Erk1/2 in AGS, MKN28, BGC823 and SGC7901 gastric cancer cell lines. In this regard, the CDK1 inhibitor p21 was activated, while cyclin D1 inactivated, after overexpression of ZIC1 in gastric cancer cells. Though the results need to be validated in future studies, our miroarray data have revealed that other key components of cell cycle kinase regulators including *TP53INPI* and *CDKN2B*, are deregulated with forced expression of ZIC1 in an individual MKN28 gastric cancer cell line (Figure 5 A and B). Therefore, we propose that ZIC1 regulates G1/S transit mainly through PI_3_K and MAPK pathways and downstream cell-cycle regulator kinases in gastric cancer cells.

Another major finding in our present study is that ZIC1 transcriptionally regulates Sonic hedgehog (Shh) signaling in gastric cancer cells. Aside from its central role on regulating gastric gland morphogenesis in human stomach, Shh signaling is also involved in the pathogenesis of gastric cancer [16,33]. Shh is frequently activated in advanced gastric adenocarcinomas and associated with aggressive tumour behavior [13,15,33]. Previous studies have shown that Shh signaling promotes the motility and invasiveness of gastric cancer cells through TGF-β-ALK5-Smad3 pathway [34]. Shh signaling could also regulate the expression of p21 and cyclin D1 in a Gli-dependent pathway [16,25]. We have observed that inhibition of Shh signaling by administration with cyclopamine suppresses AGS, BGC823 and SGC7901 gastric cancer cell migration, and regulates the expression of p21 and cyclin D1 (Figure 4). These results are consistent with previously reported studies [25,34]. Thus, we have revealed that ZIC1 plays important roles in gastric cancer progression by regulation of the Shh signaling pathway.

ZIC1 may regulate target genes in both sequence-specific and independent manners [9]. ZIC1 could regulate the transcriptional expression of targets including cyclin D1, p27, Wnt1 and Wnt7a, and modulate Notch and BMP pathways in neural development [2,9]. ZIC1 could counteract GLI by binding to GC-rich sequences, and suppress the expression of GLI-binding sequence directed reporter genes [9,35]. We identified several ZIC potential target genes in gastric cancer cells by microarray analysis. These targets are closely related to cell cycle, cell proliferation and migration (Figure 5B). The association between ZIC and downstream targets might be a clue for understanding the potential perspective of ZIC proteins in the progression of gastric cancer.

## Conclusions

Summarily, we propose a model that represents the pathways through which ZIC1 contributes to gastric cancer progression (Figure 5C). Overexpression of ZIC1 results in suppressing Hedgehog (Hh) signaling and its downstream targets including p21, p27 and cyclin D1. As a zinc finger transcription factor, ZIC1 also potentially modulates the transcriptional expression of target genes by directly binding to GC-rich sequences [2], thus functioning as a tumour suppressor by inhibition of cell proliferation, cell migration and invasion in gastric cancer.

## Methods

### Cell culture and treatment

The human gastric cancer cell lines (AGS, MKN28, BGC823 and SGC7901) were obtained from Riken Gene Bank (Japan) and American Type Culture Collection (ATCC, Manassas, VA, USA). All cell lines were cultured in RPMI 1640 medium (Invitrogen, CA, USA) supplemented with 10 % fetal bovine serum (FBS) and incubated at 5% CO_2_, 37°C and 95 % humidity. Gastric cancer cell lines (AGS, BGC823, and SGC7901) were treated with 10 μM of cyclopamine (Sigma, St Louis, MO, USA) in DMSO for 24 hours. An equivalent concentration of the vehicle (DMSO) was used as the control.

### Cell transfection

AGS, MKN28, BGC823 and SGC7901 cells were cultured for 24 h in a 6-well plate and transfected with pCDNA 3.1-ZIC1 or pCDNA 3.1 empty vector using Fugene HD (Roche) according to the manufacturer's instructions. After 48 h, the transfectants were continuously selected in RPMI 1640 medium containing G418 (200–400 μg/mL) (Merck, Germany) for 14 days.

### RT-PCR and quantitative real time PCR analysis

Total RNA (1 μg) was extracted using Trizol reagent (Invitrogen) following manufacturer’s instructions and reverse transcribed into cDNA with M-MLV RTase cDNA Synthesis Kit (Takara, Japan). The transcript levels of ZIC1 and Shh were determined by conventional RT-PCR with TaKaRa Taq polymerase (Takara, Japan) or Quantitative real-time PCR (qRT-PCR) with the SYBR Green Master Mix Kit (Takara, Japan) in an ABI 7500 PCR system. Primers used for ZIC1 were F: 5’-AAACTGGTTAACCACATCCGC and R: 5’-CTCAAACTCGCACTTGAAGG. Shh were F: 5’-GTAAGGACAAGTTGAACGCTTTG and R: 5’-GATATGTGCCTTGGACTCGTAGTA. Glyceraldehyde-3-;phosohate dehydrogenase (GAPDH) was used as an internal control. The transcript levels are expressed as 2^-ΔΔCt^ values and relative expression fold change is normalized to GAPDH.

### Cell viability assay

Cell viability was detected with a nonradioactive cell proliferation assay with 3-(4,5-dimethylthiazol-2-yl)-5-(3-carboxymethoxyphenyl)-2-(4-sulfophenyl)-2 H-tetrazolium (MTS) reagents (Promega, Madison, USA). Stable transfected cells were plated in 96-well (2000 cells/well) for 6 h, 24 h, 72 h and 120 h. The absorbance was measured at 490 nm after 1 h incubation with CellTiter 96 Aqueous One Solution reagent.

### Cell-cycle and cell apoptosis analysis

Cell cycle distributions were detected by the flow cytometry analysis. Cells transfected with pCDNA3.1-ZIC1 or pCDNA3.1 empty vector were harvested and washed with PBS. Cellular DNA was stained with cell cycle staining solution containing propidium iodide (PI) at 4 °C in dark. Cell cycle was determined using a FACS Calibur and analyzed with the ModFitLT software (Phoenix, USA).

Cell apoptosis was performed using FITC Annexin V Apoptosis Detection Kit II (BD Pharmingen) by flow cytometry analysis. Transiently transfected cells were suspended in Annexin V Binding Buffer. Then FITC Annexin V and PI solutions were added in sequence. After incubation for 15 min, the stained cells were analyzed by flow FACScan flow cytometry (Becton Dickinson, USA).

### Cell migration and invasion assays

Cell migration was assessed by modified Boyden transwell chambers assay (Coring, USA). Briefly, cells were cultured in serum-free medium for 24 h and 5 × 10^4^ cells were plated to the upper chamber in 300 μL medium containing 5 % FBS. After 16 h of incubation, non-migratory cells in the upper chamber were carefully removed with a cotton swab. Migrated Cells were stained with DAPI Staining Solution. The cell numbers were randomly counted in five fields (×400 magnification).

Cell invasion was performed in a millipore 24-well coated with BD Matrigel. After starvation in serum-free medium for 24 h, 1 × 10^5^ cells were plated to the upper chamber in 300 μL of medium containing 5 % FBS, while the lower chamber was filled with 600 μL of culture medium with 15 % FBS. After having been incubated for 30 h, the membranes were incubated with Cell Stain Solution (Millorpore, USA). The dye mixture was washed by Extraction Buffer and transferred to a 96-well for colorimetric measurement at 560 nm.

### Western blot analysis

Total proteins were extracted from cells using radio-immunoprecipitation assay lysis buffer supplemented with protease inhibitor. Lysates were resolved on 6-12 % SDS-PAGE minigels and transferred to PVDF membranes (Millipore, Bedford, MA). Membranes were blocked in 5 % milk with TBST and incubated in 4°C overnight with following antibodies: ZIC1 (1:500; Abcam), phospho-Akt (1:1000; Cell Signaling), Akt (1:500; Abcam), phospho-Erk1/2 (1:1000; Cell Signaling), Erk1/2 (1:1000; Cell Signaling), p21^Waf1/Cip1^ (1:1000; Cell Signaling), p27 ^Kip1^ (1:500; Epitomics), cyclin D1 (1:1000; Cell Signaling) and Shh (1:1000; Cell Signaling). Accordingly, secondary antibodies coupled to horseradish peroxidise (HRP) were visualized using a chemiluminescence with Las-4000 Imaging System (Fujifilm, Japan). The relative densities of proteins were quantified with Image J. software and normalized to β-actin (1:2500, Multisciences Biotech).

### cDNA microarray analysis

Total RNA was isolated from MKN28 cells which stably transfected with pCDNA3.1-ZIC1 or pCDNA3.1 empty vector, and was reverse transcribed to cDNA. Labeled samples were hybridized with Agilent whole human genome containing more than 41,000 probes (http://www.ncbi.nlm.nih.gov/geo/query/acc.cgi?acc=GSE38924). The microarray data were analyzed using Agilent Feature Extraction software. We selected a fold change ≥1.5 or ≤ −1.5 as a significant difference.

### Statistical analysis

Student’s *t* test was performed to compare two-independent data, while Chi-square or fisher exact test was used to analyze categorical variables. A cut-off of p < 0.05 was applied for statistical significance.

## Competing Interests

The authors confirm that there are no conflicts of interest.

## Author’s contributions

JZ performed the experiments, analysis the data, and wrote the paper ; SJC performed the experiments and analysis the data; MX performed the experiments; QD Contributed reagents/materials/analysis tool; JTC Contributed reagents/materials/ analysis tool; HCJ Conceived and designed the experiments, contributed reagents and materials; JMS Conceived and designed the experiments, contributed reagents and materials; LJW Conceived , designed, and performed the experiments, analysis the data, , contributed reagents and materials and wrote the paper.

## Pre-publication history

The pre-publication history for this paper can be accessed here:

http://www.biomedcentral.com/1471-2407/12/290/prepub

## Supplementary Material

Additional file 1 ** Figure S1.** ZIC1 suppresses gastric cancer cell invasion. A BD Matrigel coated chamber was used to assess cell invasion. 1 × 10^5^ AGS cells stably transfected with pCDNA3.1-ZIC1 or pCDNA3.1 empty vector were plated to the upper chamber and incubated for 24 h. Invaded cells were stained with Cell Stain solution, and detected on a standard microplate reader (560 nm). The relative invaded cells are expressed as the percentage rate compared with pcDNA3.1 empty vector transfectants (Bars, ±S.D. ; *p < 0.05). Click here for file

Additional file 2 ** Figure S2.** ZIC1 does not affect the cell apoptotic activity in gastric cancer cells. The cell apoptosis rate was determined by the Annexin V-PI flowcytometry assay after transient transfection with pCDNA3.1-ZIC1 or pCDNA3.1 empty vector in AGS and MKN28 cells for 24 h. Region **LR** indicates the percentage of early apoptotic cells, **UR** shows late apoptotic cells. Click here for file

Additional file 3 ** Figure S3.** Overexpression of ZIC1 inhibits the expression of Shh mRNA. AGS and SGC7901 cells were stably transfected with pCDNA3.1-ZIC1 or empty vector pCDNA3.1. The expression levels of ZIC1 and Shh mRNA were performed by RT-PCR. GAPDH was used as an internal control. Click here for file

## References

[B1] Aruga J Yokota N Hashimoto M Furuichi T Fukuda M Mikoshiba K A novel zinc finger protein, zic, is involved in neurogenesis, especially in the cell lineage of cerebellar granule cells J Neurochem 1994 63 5 1880 1890 7931345 10.1046/j.1471-4159.1994.63051880.x

[B2] Merzdorf CS Emerging roles for zic genes in early development Dev Dyn 2007 236 4 922 940 10.1002/dvdy.21098 17330889

[B3] Brill E Gobble R Angeles C Lagos-Quintana M Crago A Laxa B Decarolis P Zhang L Antonescu C Socci ND ZIC1 overexpression is oncogenic in liposarcoma Cancer Res 2010 70 17 6891 6901 10.1158/0008-5472.CAN-10-0745 20713527 PMC3266950

[B4] Pourebrahim R Van Dam K Bauters M De Wever I Sciot R Cassiman JJ Tejpar S ZIC1 gene expression is controlled by DNA and histone methylation in mesenchymal proliferations Febs Lett 2007 581 26 5122 5126 10.1016/j.febslet.2007.09.061 17936758

[B5] Wong YF Cheung TH Lo KW Yim SF Siu NS Chan SC Ho TW Wong KW Yu MY Wang VW Identification of molecular markers and signaling pathway in endometrial cancer in Hong Kong Chinese women by genome-wide gene expression profiling Oncogene 2007 26 13 1971 1982 10.1038/sj.onc.1209986 17043662

[B6] Yokota N Aruga J Takai S Yamada K Hamazaki M Iwase T Sugimura H Mikoshiba K Predominant expression of human zic in cerebellar granule cell lineage and medulloblastoma Cancer Res 1996 56 2 377 383 8542595

[B7] Gan L Chen S Zhong J Wang X Lam EK Liu X Zhang J Zhou T Yu J Si J ZIC1 is downregulated through promoter hypermethylation, and functions as a tumor suppressor gene in colorectal cancer PLoS One 2011 6 2 e16916 10.1371/journal.pone.0016916 21347233 PMC3039653

[B8] Wang LJ Jin HC Wang X Lam EK Zhang JB Liu X Chan FK Si JM Sung JJ ZIC1 is downregulated through promoter hypermethylation in gastric cancer Biochem Biophys Res Commun 2009 379 4 959 963 10.1016/j.bbrc.2008.12.180 19135984

[B9] Aruga J The role of Zic genes in neural development Mol Cell Neurosci 2004 26 2 205 221 10.1016/j.mcn.2004.01.004 15207846

[B10] Danaei G Vander HS Lopez AD Murray CJ Ezzati M Causes of cancer in the world: comparative risk assessment of nine behavioural and environmental risk factors Lancet 2005 366 9499 1784 1793 10.1016/S0140-6736(05)67725-2 16298215

[B11] Hohenberger P Gretschel S Gastric cancer Lancet 2003 362 9380 305 315 10.1016/S0140-6736(03)13975-X 12892963

[B12] Wu WK Cho CH Lee CW Fan D Wu K Yu J Sung JJ Dysregulation of cellular signaling in gastric cancer Cancer Lett 2010 295 2 144 153 10.1016/j.canlet.2010.04.025 20488613

[B13] Ma X Chen K Huang S Zhang X Adegboyega PA Evers BM Zhang H Xie J Frequent activation of the hedgehog pathway in advanced gastric adenocarcinomas Carcinogenesis 2005 26 10 1698 1705 10.1093/carcin/bgi130 15905200

[B14] Echelard Y Epstein DJ St-Jacques B Shen L Mohler J McMahon JA McMahon AP Sonic hedgehog, a member of a family of putative signaling molecules, is implicated in the regulation of CNS polarity Cell 1993 75 7 1417 1430 10.1016/0092-8674(93)90627-3 7916661

[B15] Katoh Y Katoh M Hedgehog signaling pathway and gastric cancer Cancer Biol Ther 2005 4 10 1050 1054 10.4161/cbt.4.10.2184 16258256

[B16] Saqui-Salces M Merchant JL Hedgehog signaling and gastrointestinal cancer Biochim Biophys Acta 2010 1803 7 786 795 10.1016/j.bbamcr.2010.03.008 20307590 PMC5340308

[B17] Maurus D Harris WA Zic-associated holoprosencephaly: zebrafish Zic1 controls midline formation and forebrain patterning by regulating Nodal, Hedgehog, and retinoic acid signaling Genes Dev 2009 23 12 1461 1473 10.1101/gad.517009 19528322 PMC2701571

[B18] Aruga J Tohmonda T Homma S Mikoshiba K Zic1 promotes the expansion of dorsal neural progenitors in spinal cord by inhibiting neuronal differentiation Dev Biol 2002 244 2 329 341 10.1006/dbio.2002.0598 11944941

[B19] Merzdorf CS Sive HL The zic1 gene is an activator of Wnt signaling Int J Dev Biol 2006 50 7 611 617 10.1387/ijdb.052110cm 16892174

[B20] Aruga J Inoue T Hoshino J Mikoshiba K Zic2 controls cerebellar development in cooperation with Zic1 J Neurosci 2002 22 1 218 225 11756505 10.1523/JNEUROSCI.22-01-00218.2002 PMC6757594

[B21] Sharma PS Sharma R Tyagi R Inhibitors of cyclin dependent kinases: useful targets for cancer treatment Curr Cancer Drug Targets 2008 8 1 53 75 10.2174/156800908783497131 18288944

[B22] Arici DS Tuncer E Ozer H Simek G Koyuncu A Expression of retinoblastoma and cyclin D1 in gastric carcinoma Neoplasma 2009 56 1 63 67 10.4149/neo_2009_01_63 19152247

[B23] Alao JP The regulation of cyclin D1 degradation: roles in cancer development and the potential for therapeutic invention Mol Cancer 2007 6 24 17407548 10.1186/1476-4598-6-24 PMC1851974

[B24] Abbas T Dutta A p21 in cancer: intricate networks and multiple activities Nat Rev Cancer 2009 9 6 400 414 10.1038/nrc2657 19440234 PMC2722839

[B25] Ohta M Tateishi K Kanai F Watabe H Kondo S Guleng B Tanaka Y Asaoka Y Jazag A Imamura J p53-Independent negative regulation of p21/cyclin-dependent kinase-interacting protein 1 by the sonic hedgehog-glioma-associated oncogene 1 pathway in gastric carcinoma cells Cancer Res 2005 65 23 10822 10829 10.1158/0008-5472.CAN-05-0777 16322228

[B26] Park JM Xian XS Choi MG Park H Cho YK Lee IS Kim SW Chung IS Antiproliferative mechanism of a cannabinoid agonist by cell cycle arrest in human gastric cancer cells J Cell Biochem 2011 112 4 1192 1205 10.1002/jcb.23041 21312237

[B27] Xie K Abbruzzese JL Developmental biology informs cancer: the emerging role of the hedgehog signaling pathway in upper gastrointestinal cancers Cancer Cell 2003 4 4 245 247 10.1016/S1535-6108(03)00246-0 14585350

[B28] Taipale J Chen JK Cooper MK Wang B Mann RK Milenkovic L Scott MP Beachy PA Effects of oncogenic mutations in Smoothened and Patched can be reversed by cyclopamine Nature 2000 406 6799 1005 1009 10.1038/35023008 10984056

[B29] Sabater L Bataller L Suarez-Calvet M Saiz A Dalmau J Graus F ZIC antibodies in paraneoplastic cerebellar degeneration and small cell lung cancer J Neuroimmunol 2008 201-202 163 165 18639938 10.1016/j.jneuroim.2008.01.018 PMC2582201

[B30] Chambard JC Lefloch R Pouyssegur J Lenormand P ERK implication in cell cycle regulation Biochim Biophys Acta 2007 1773 8 1299 1310 10.1016/j.bbamcr.2006.11.010 17188374

[B31] Arcaro A Guerreiro AS The phosphoinositide 3-kinase pathway in human cancer: genetic alterations and therapeutic implications Curr Genomics 2007 8 5 271 306 10.2174/138920207782446160 19384426 PMC2652403

[B32] de Carcer G Perez DCI Malumbres M Targeting cell cycle kinases for cancer therapy Curr Med Chem 2007 14 9 969 985 10.2174/092986707780362925 17439397

[B33] van den Brink GR Hardwick JC Tytgat GN Brink MA Ten KF Van Deventer SJ Peppelenbosch MP Sonic hedgehog regulates gastric gland morphogenesis in man and mouse Gastroenterology 2001 121 2 317 328 10.1053/gast.2001.26261 11487541

[B34] Yoo YA Kang MH Kim JS Oh SC Sonic hedgehog signaling promotes motility and invasiveness of gastric cancer cells through TGF-beta-mediated activation of the ALK5-Smad 3 pathway Carcinogenesis 2008 29 3 480 490 18174246 10.1093/carcin/bgm281

[B35] Koyabu Y Nakata K Mizugishi K Aruga J Mikoshiba K Physical and functional interactions between Zic and Gli proteins J Biol Chem 2001 276 10 6889 6892 10.1074/jbc.C000773200 11238441

