# Supplementary figures and images for: ZIC1 modulates cell-cycle distributions and cell migration through regulation of sonic hedgehog, PI3K and MAPK signaling pathways in gastric cancer
Source: BMC Cancer. 2012 Jul 16;12:290. doi: 10.1186/1471-2407-12-290 (PMC3411411; doi:10.1186/1471-2407-12-290)

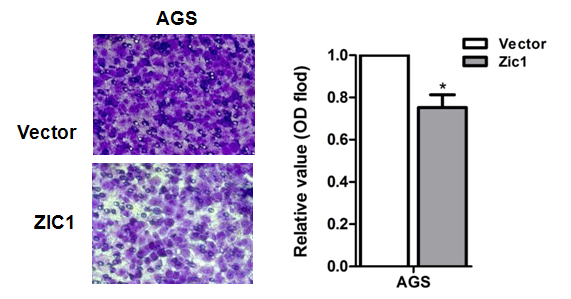

Supplement: Additional file 1 — Figure S1. ZIC1 suppresses gastric cancer cell invasion. A BD Matrigel coated chamber was used to assess cell invasion. 1 × 10^5^ AGS cells stably transfected with pCDNA3.1-ZIC1 or pCDNA3.1 empty vector were plated to the upper chamber and incubated for 24 h. Invaded cells were stained with Cell Stain solution, and detected on a standard microplate reader (560 nm). The relative invaded cells are expressed as the percentage rate compared with pcDNA3.1 empty vector transfectants (Bars, ±S.D. ; *p < 0.05). [file 1471-2407-12-290-S1.tiff]

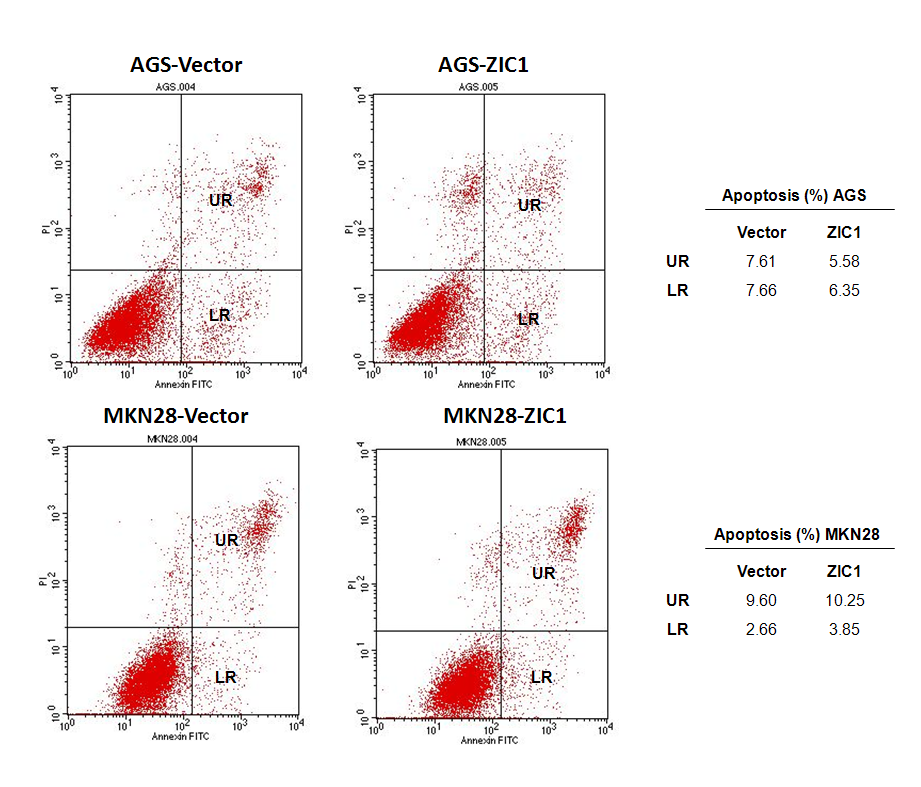

Supplement: Additional file 2 — Figure S2. ZIC1 does not affect the cell apoptotic activity in gastric cancer cells. The cell apoptosis rate was determined by the Annexin V-PI flowcytometry assay after transient transfection with pCDNA3.1-ZIC1 or pCDNA3.1 empty vector in AGS and MKN28 cells for 24 h. Region LR indicates the percentage of early apoptotic cells, UR shows late apoptotic cells. [file 1471-2407-12-290-S2.tiff]

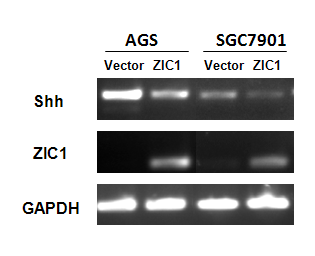

Supplement: Additional file 3 — Figure S3. Overexpression of ZIC1 inhibits the expression of Shh mRNA. AGS and SGC7901 cells were stably transfected with pCDNA3.1-ZIC1 or empty vector pCDNA3.1. The expression levels of ZIC1 and Shh mRNA were performed by RT-PCR. GAPDH was used as an internal control. [file 1471-2407-12-290-S3.tiff]
